# Influence of Feeding Substrates on the Presence of Toxic Metals (Cd, Pb, Ni, As, Hg) in Larvae of *Tenebrio molitor*: Risk Assessment for Human Consumption

**DOI:** 10.3390/ijerph16234815

**Published:** 2019-11-30

**Authors:** Cristina Truzzi, Silvia Illuminati, Federico Girolametti, Matteo Antonucci, Giuseppe Scarponi, Sara Ruschioni, Paola Riolo, Anna Annibaldi

**Affiliations:** 1 Dipartimento di Scienze della Vita e dell’Ambiente, Università Politecnica delle Marche, via Brecce Bianche, 60131 Ancona, Italy; f.girolametti@pm.univpm.it (F.G.); matteo.antonucci.89@outlook.com (M.A.); g.scarponi@univpm.it (G.S.); a.annibaldi@univpm.it (A.A.); 2 Dipartimento di Scienze Agrarie, Alimentari ed Ambientali, Università Politecnica delle Marche, via Brecce Bianche, 60131 Ancona, Italy; s.ruschioni@univpm.it (S.R.); p.riolo@univpm.it (P.R.)

**Keywords:** edible insects, *Tenebrio molitor*, food chemical safety, toxic metals, mercury-selenium balance, atomic absorption spectrometry, direct mercury analyzer

## Abstract

Larvae of *Tenebrio molitor* L. (Coleoptera: Tenebrionidae) are particularly suitable as novel food for the human consumption; nevertheless, there are some potential safety risks linked with insect consumption. In this study we investigated the presence of Cd, Pb, Ni, As, Hg in new feeding substrates coming from solid residues generated by olive fruits processing, called olive-pomace, and their influence on the metal content in larvae of *T. molitor*. Morover, bioaccumulation factor and the mercury-selenium balance were evaluated. Analyses were carried out via graphite furnace atomic absorption spectrophotometry for Cd, Pb, Ni, As and Se, and via Direct Mercury Analyzer for Hg. All metal concentrations found in feeding substrates were below the legal limit of undesirable substances in animal feed (2002/32/EC). Concentrations in larvae were in the range (mg kg^−1^ wet weight): Cd 0.008–0.016, Pb 0.063–0.079, Ni 0.03–0.63, As 0.021–0.023, Hg 0.12 × 10^−3^–0.49 × 10^−3^, and Se 0.057–0.085. Statistically significant correlation between metal content in feeding substrates and in larvae was evidenced only for Hg, which bioaccumulates. Se protects from mercury toxicity, with a Selenium Health Benefit Value (HVB_Se_) of > 0. Overall, our results indicate that the risk of exposure to metals from consumption of mealworm larvae is relatively low and in compliance with European Union regulations.

## 1. Introduction

Due to the rapid increase in world population, the waste of food and resources, and unsustainable food production practices, the use of alternative food sources is currently strongly promoted [1]. In this perspective, insects may represent a valuable alternative to main animal food sources since insects grow and reproduce easily, have a high feed conversion efficiency, and can transform low value of growing substrates into high value food resource [2,3,4]. Moreover, insects have a high nutrition potential due to an interesting amino acids composition, an adequate content of minerals, vitamins and polyunsaturated fatty acids [5,6,7]. Nevertheless, the nutritional value of edible insects is highly variable, and the differences may also depend on their feeding substrates [8,9]. Moreover, there are some potential safety risks linked with insect consumption: insects, similarly to other animal products, might accumulate hazardous chemicals, such as heavy metals [10,11,12], dioxins [13], and flame retardants [14,15]. For centuries insects have been used as food [16], but in Europe, insect make up only 2% of the human foodstuff [17]. Most likely, the possible acceptance and the change in the approach of developed countries with regards to entomophagy would certainly be supported by food chemical safety [18]. Recently, the European Union introduced a new regulation about the so-called novel foods, namely Regulation (EU) 2015/2283 of the European Parliament and of the Council of 25 November, 2015, which has been applied from 1 January, 2018. This legislation concerns the reviewing, clarification and updating of the categories of novel foods, which now include whole insects and their parts. Hence, in depth studies on specified insect species aimed to define their safety for possible mass production, are becoming even more important.

Recently, the European Food Safety Authority (EFSA) proposed a list of insect species with the greatest potential to be used as food and feed in the EU [8], whiich includes the yellow mealworm, *Tenebrio molitor* L. (Coleoptera: Tenebrionidae). The yellow mealworm is particularly efficient in converting organic substance and it is receiving increasing attention as a “bio-converter” [1], as it can transform diet substrate with a low nutritional value in a rich protein product. Moreover, it is characterized by high nutritional value [19], comparable with those of beef and chicken. Its larvae are particularly suitable for human consumption [19,20,21], and it is a perfect candidate for rearing with the aim of producing a novel food for the future. Nevertheless, nutritional value and potential safety risks linked with insect consumption depend from the feeding substrate used to breed insects [8], as insects can accumulate toxic metals from their feeding substrates and transfer them in insect-based food products [22]. Different studies on the yellow mealworm larvae, for example, showed that the insects accumulate cadmium, lead, and arsenic in their bodies when they feed on organic matter in soils spiked with these metals [23].

In the framework of the project “Edible insects: new frontiers in food—FoodIN”, by the Politechnic University of Marche, new feeding substrates for rearing *T*. *molitor*, based on different mixtures of organic wheatmeal and organic olive-pomace, were investigated, in order to obtain a production of large amounts of low-cost, nutritious, healthy and safe insect-based foods. Olive oil represents a traditional product of high economic significance for most of Mediterranean countries. However, fruits processing generates solid residues, called olive-pomace, which represent a severe environmental issue, mainly due to the enormous volumes generated [24]. Therefore, several valorisation options have been explored on the use of waste and by-products from the olive production as animal feed, in the framework of sustainable circular economy [25,26,27,28]. 

Within this project, the objectives of the present investigation are the following: (i) to determine the concentration of Cd, Pb, Hg, Ni and As in mealworm larvae reared on substrates made up of organic wheat milling and olive processing by-products (these metals are considered dangerous and priority pollutants by the regulation in force (European Parliament and Council of European Union, Directive 2000/60/EC), (ii) to study the influence of feeding substrates on the presence of heavy metals and Se in larvae of *T. molitor* by exploring the concentrations of these elements in feeding substrates and in frass, (iii) to discuss human exposure risks with regards to international food safety regulations. At this regard, while for Cd and Pb the risks for human is associated to food consumption, it is known that Selenium may protect against the toxic effects of mercury, forming insoluble and/or biologically unavailable HgSe complexes, with a consequent reduction of the risk associated with the ingestion of Hg-contaminated food [29,30,31,32,33]. So, the Se concentration was also determinated, to evaluate the risk assessment for the human consumption of larvae of *T. molitor* associated to Hg-intake.

## 2. Materials and Methods 

### 2.1. Insect Colony Maintenance

*Tenebrio molitor* larvae were purchased by a commercial pet shop PlanetFish&Co. (Ancona, Italy). Insects were reared in plastic boxes (400 × 300 × 60 mm L × W × H) and fed with organic wheatmeal (Molino del Conero, San Biagio, Osimo, Italy). Organic carrots were used to provide moisture. Pupae were collected and placed in plastic boxes (200 × 150 × 60 mm, L × W × H) until the emergence of adults. Newly emerging adults were placed in plastic containers lined with filter papers (Whatman, Dassel, Germany), and supplied with organic wheatmeal and carrots. Every four days, the eggs, which glued to the filter paper, were recovered, placed in plastic containers (200 × 150 × 60 mm, L × W × H), and monitored until the first instar hatched. The insect colony was maintained in a climatic chamber at 28 ± 1 °C, 60 ± 5% RH and a 24 h dark photoperiod.

### 2.2. Insect Feeding Substrates

Based on local availability, organic wheat flour and organic wheatmeal (Molino del Conero, San Biagio, Osimo (AN), Italy), used as control feeding substrates, and organic olive-pomace (Azienda Agricola “I tre filari”, Recanati (AN), Italy) were selected as ingredients for the formulation of the feeding substrates. Five experimental diets were tested: (a) 100% organic wheat flour, (b) 100% organic wheatmeal with increasing, (c) 25%, (d) 50% and (e) 75% content of organic olive-pomace.

### 2.3. Insect Rearing

For each experimental diet, 4000 first instar larvae (no. 3 repicates for each diet) were placed in plastic containers (55 × 36 × 15 cm), fed with 0.25 g/larva of feeding substrate and supplied with 0.05 g/larva of organic carrots as an additional source of water [34,35,36]. Feeding substrates (0.125 g/larva) were added every two weeks while organic carrots (0.05 g/larva) were added twice for a week. Each time, the larvae had been allowed access to carrot pieces for two hours. The vegetable residues were then removed in order to reduce the feeding substrate contamination. No antibiotics were used for the treatment of insects. Larvae were reared in a climatic chamber at 28 ± 1°C, 60 ± 5% RH and 0:24 h (L:D) photoperiod [37], for about 4 months (from first instar to last instar). Samples of lyophilized freeze-killed last instar larvae, diets and frass (composed by excrements, feeding substrate residues and exuviae) were placed into plastic bags and stored at −20 °C until chemical analyses.

### 2.4. Laboratory and Apparatus

A clean room laboratory ISO 14644–1 Class 6, with areas at ISO Class 5 under laminar flow, was used for all laboratory activities. Samples were handled with plastic materials (low density polyethylene 30 mL cylindrical containers, Kartell, Milan, Italy, Mod K912), washed with acid-cleaning procedures and rinsed with Milli-Q water obtained from a two-stage system Midi (Elix and Milli-Q) from Millipore (Bedford, MA, USA), in order to avoid any metal contamination [38]. The laboratory analytical balance was the AT261 Mettler Toledo (Greifensee, Switzerland, readability 0.01 mg, repeatability SD = 0.015 mg). Variable volume micropipettes and neutral tips were from Brand (Wertheim, Germany, Transferpette). 

### 2.5. Chemical Analyses and Quality Control

Samples were minced, homogenized (homogenizer MZ 4110, DCG Eltronic), and divided in aliquot of 0.5 g each. To determine the moisture, samples were accurately weighed and freeze-dried (Edwards EF4 modulyo, Crawley, Sussex, England) until constant weight (±0.2 mg). Analyses were carried out on three aliquots per sample. For the determination of Cd, Pb, Ni, As and Se, samples were digested in a high-quality (65% w/v) nitric acid HNO_3_ and 30% v/v H_2_O_2_ (Merk) mixture in a Microwave Accelerated Reaction System, MARS-X, 1500 W (CEM, Mathews, NC, USA). The operational parameters were divided in three steps. For each step, the oven power was set to 800 W, and the magnetron power to 100%, time to reach settings in 10 min, and a hold time of 5 min. Pressure and temperature were set to 50 psi and 150 °C for the first step, 90 psi and 160 °C for the second step, and 150 psi and 175 °C for the third step; cooling time was set to 15 min. 

Quantitative determinations Cd, Pb, Ni, As and Se were made with an Agilent DUO 240FS atomic absorption spectrometer (Agilent, Santa Clara, CA 95051, USA) equipped with graphite furnace (GTA120 Graphite Tube Atomizer) and with Zeeman-effect background corrector. Atomic absorption spectrometry standard solutions for Cd, Pb, Ni, As, Hg and Se (Titrisol grades from Merck) were used to build up the calibration curves. Argon (99.998% purity) was used as carrier gas. Hollow cathode lamps were used as a light source. Cadmium, lead, arsenic, nickel and selenium were measured at wavelengths of 228.8, 283.3, 193.7, 232.0, 196.0. To improve the analytical measurements a 0.2% Pd matrix modifier in citric acid was used. Procedural blanks accounted for less than 1% of the total element concentrations in samples.

The total mercury content was quantified by thermal decomposition amalgamation atomic absorption spectrometry (TDA AAS) [39] using a Direct Mercury Analyzer (DMA-1, Milestone, Sorisole, BG, Italy). The homogenised samples were weighed directly into quartz containers, then heated using compressed air (purity 99.998%) as the oxidant gas. The Hg vapours pass through a catalyst, and the products of combustion are then removed and trapped in a gold amalgamator. High temperatures (850 °C) are applied for desorption, and the Hg content quantified by determining the absorption at 253.7 nm. We optimized the analytical method for mercury determination in *T. molitor* larvae and in feed. The optimal reading conditions in the mercury analyzer were the following: sample drying temperature, 200 or 250 °C (for yellow mealworm and feeding substrate, respectively) for 60 s; decomposition temperature, 650 °C for 120 s; desorbption temperature, 650 °C for 60 s; absorbance determined at 253.7 nm. The ranges for the detection cells of the equipment were: 0.03 to 200 ng and 200 to 1500 ng. It was not possible to perform frass analysis with DMA-1 because of the presence of some substances that rapidly altered and destroyed the catalytic tube. Calibration curve technique was used for the quantification of mercury content [40]. To correct for possible mercury contamination during the analysis, the mercury concentration of a blank was subtracted from sample Hg concentrations. All analyses were carried out in triplicate.

Analytical quality control was achieved using the certified reference material, DORM-2 Dogfish muscle (National Research Council of Canada). Table 1 shows the validation parameters for the analytical procedures. Results were in good agreement with the certified values, and the standard deviation were low, proving good repeatability of the methods.

### 2.6. Bioaccumulation Factor and Risk Assessment Analyses

The bioaccumulation factor (BAF) was calculated on a dry weight (dw) basis [41], as BAF = concentration in the organism/concentration in the feed provided. Thus, a BAF greater than 1 suggests bioaccumulation of the element from the substrate into the insect.

The mercury–selenium balance was studied using the Selenium Health Benefit Value (HBV_Se_) [42]. In order to reflect the amount of physiological Se that is potentially provided or lost with respect to sequestration by the associated Hg, the relative amount of Se available is:

([Se − Hg]/Se).(1)

This amount (Equation (1)) is multiplied by the total amount of Hg and Se present in the food (Se + Hg). So, the following equation was used:

HBV_Se_ = ([Se − Hg]/Se) × (Se + Hg), (2)

where Se and Hg represent the total content of Se and Hg in mg∙kg^−1^. A positive sign of this parameter would negate risks associated with Hg toxicity.

### 2.7. Data Treatment and Statistical Analysis

Data are expressed as mean ± standard deviation (SD). To compare metals and selenium concentrations between samples, the one-way-Analysis of Variance (ANOVA test), followed by the Multiple Range Test [43] was performed after testing the homogeneity of variance with Levene’s test. Significant differences were evaluated at the 95% confidence level. When the ANOVA test gave a *p*-value equal to 0.0000, in the text it was indicated as *p* < 0.0001. All statistical treatments were performed using Statgraphics 18 Centurion (2017) [44].

## 3. Results and Discussion

### 3.1. Cadmium

**Figure 1:**
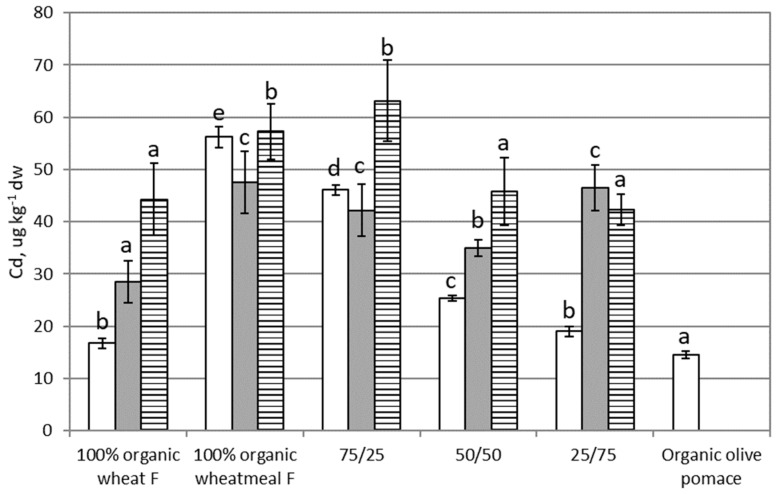
Cadmium content (as µg∙kg^−1^ dw) in feeding substrates and in organic olive-pomace (white bars), *T. molitor* larvae (grey bars), and frass (black and white bars). Each bar represents the mean ± standard deviation of at least three measurements. Feeding substrates: 100% organic wheat flour; 100% organic wheatmeal; 75/25: 75% organic wheatmeal and 25% organic olive-pomace; 50/50: 50% organic wheatmeal and 50% organic olive-pomace; 25/75: 25% organic wheatmeal and 75% organic olive-pomace. Different letters within the same matrix indicate statistically significant differences among experimental groups (*p* < 0.05). (a) 100% organic wheat flour, (b) 100% organic wheatmeal with increasing, (c) 25%, (d) 50% and (e) 75% content of organic olive-pomace.

Figure 1 shows results about Cd content in feeding substrates, larvae of *T. molitor*, and frass. Cd content in feeding substrates varied from 18 to 56 µg∙kg^−1^ dw. In particular, 100% organic olive-pomace feeding substrate showed the lowest Cd content, whereas 100% organic wheatmeal showed the highest concentration. With the reduction in the percentage of organic wheatmeal in feeding substrates and the simultaneous increase in the percentage of organic olive-pomace, a statistically significant reduction of Cd content were evidenced (*p* < 0.0001). Referring the EC limit (2002/32/EC) on undesirable substances in animal feed, the legal limit for Cd in complete feed is 0.5 mg∙kg^−1^ (maximum content relative to a feedingstuff with a moisture content of 12%). Cd content in tested feeding substrates (calculated for a moisture content of 12%) was from 0.015 to 0.05 mg∙kg^−1^, i.e., from 10 to 30-fold lower than the legal limit. Then, tested feeding substrates were safe from the point of view of Cd content.

Cd content in larvae (from 28 to 46 µg∙kg^−1^ dw) was of the same order of feeding substrates, and larvae reared on organic wheat flour showed a content significantly lower than larvae reared on other substrates (*p* < 0.0001). No statistically significant correlation was found between Cd content in feeding substrates and in larvae (r = 0.5988, *p* = 0.2860); therefore, the content of Cd in larvae is not influenced by Cd content in the feeding substrate. The BAF for Cd was next to 1 (Table 2), indicating that Cd did not bioaccumulate in larvae of *T. molitor*.

Cd content in frass showed a moderately strong relationship statistically significant with the corresponding feeding substrate (r = 0.8986, *p* = 0.0381), indicating that the model as fitted explains 81% of the variability of Cd in frass. Cd content in frass was generally higher than in the corresponding larvae, confirming that this element is not retained by this specie. From these results, consistent with literature data [41] we can conclude that Cd present in the feeding substrate penetrates in the body of larvae and was then excreted without bioaccumulation.

### 3.2. Lead

**Figure 2:**
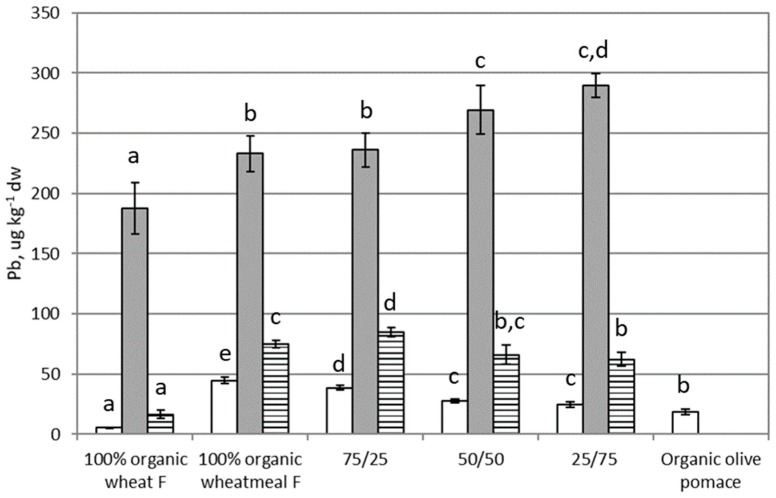
Lead content (as µg∙kg^−1^ dw) in feeding substrates and in organic olive-pomace (white bars), *T. molitor* larvae (grey bars), and frass (black and white bars). Each bar represents the mean ± standard deviation of at least three measurements. Feeding substrates: 100% organic wheat flour; 100% organic wheatmeal; 75/25: 75% organic wheatmeal and 25% organic olive-pomace; 50/50: 50% organic wheatmeal and 50% organic olive-pomace; 25/75: 25% organic wheatmeal and 75% organic olive-pomace. Different letters within the same matrix indicate statistically significant differences among experimental groups (*p* < 0.05).

Figure 2 shows results on Pb content in feeding substrates, larvae of *T. molitor*, and frass. Pb content in feeding substrates varied from 5 to 45 µg∙kg^−1^ dw. In particular, organic wheat flour showed the lowest Pb content, whereas organic wheatmeal showed the highest concentration. With the reduction in the percentage of organic wheatmeal in feeding substrates and the simultaneous increase in the percentage of organic olive-pomace, a statistically significant reduction of Pb content were evidenced (*p* < 0.0001). Referring the EC limit (2002/32/EC) on undesirable substances in animal feed, the legal limit for Pb in complete feed is 5 mg∙kg^−1^ (maximum content relative to a feedingstuff with a moisture content of 12 %). Pb content in tested feeding substrates (calculated for a moisture content of 12%) was from 0.005 to 0.039 mg∙kg^−1^, i.e., from 130 to 1000-fold lower than the legal limit. Tested feeding substrates were absolutely safe from the point of view of Pb content.

Pb content in larvae (from 190 to 290 µg∙kg^−1^ dw) was very higher with respect to Pb content in feeding substrates. Statistically significant differences (*p* = 0.0004) in Pb content were observed between larvae reared on different substrates, with the lowest concentration in larvae reared on 100% organic wheat flour, and the highest in larvae reared on feeding substrate composed by 25% organic whetmeal and 75% organic olive-pomace. No statistically significant correlation was found between the Pb content in substrates and in larvae (r = 0.3496, *p* = 0.5641); therefore, it seems that Pb content in larvae is not influenced by the Pb content in the feeding substrate, as demonstrated also by van der Fels-Klerx (2016) [41]. On the other hand, the high BAF values (from 5 to 34) (Table 2), tend for a bioaccumulation of this metal. On the light of these results, we hypothesized that the high levels of Pb in insects could be due to an additional source, such as organic carrots, which were supplied to insect as water source. Root vegetables have a great ability to accumulate heavy metals. The EU commission regulation No 1881/2006 of 19 December 2006 and amending Regulations No 420/2011 of 29 April 2011 establish a maximum level of lead in vegetables at 0.1 mg∙kg^−1^ ww. In general, carrots show a Pb concentration higher than the legal limit: the edible part of carrots from contaminated soil contained Pb levels of: ~0.23 mg∙kg^−1^ ww from China, 1.8–2.5 mg∙kg^−1^ ww from UK, 1.3–28 mg∙kg^−1^ ww from Germany [45]. In the un-contaminated soil of USA, carrots contained 0.2–0.3 mg∙kg^−1^ ww [46]. These data suggest that most likely carrots used in this study could have a Pb content higher than tested feeding substrates (these last from 5 to 40 µg∙kg^−1^ ww), contributing to a major part in larvae contamination.

Pb content in frass varied from 17 to 85 µg∙kg^−1^ dw. Statistically significant differences were evidenced between frass coming from different feeding substrates (*p* < 0.0001). Frass showed a content of Pb from two to three-fold higher than the corresponding feeding substrate and a statistically significant correlation was found between Pb content in frass and in feeding substrate (r = 0.9312, *p* = 0.0214), underlaying as Pb content in frass is influenced by Pb content in the feeding substrate [41].

### 3.3. Nickel

**Figure 3:**
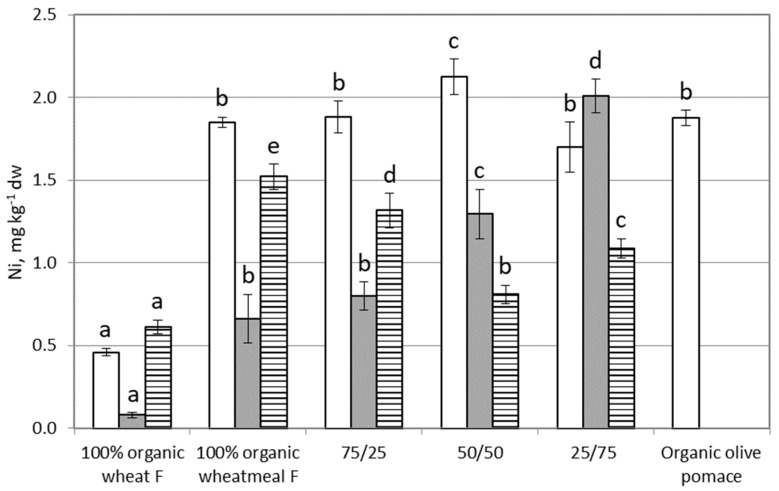
Nickel content (as mg∙kg^−1^ dw) in feeding substrates and in organic olive-pomace (white bars), *T. molitor* larvae (grey bars), and frass (black and white bars). Each bar represents the mean ± standard deviation of at least three measurements. Feeding substrates: 100% organic wheat flour; 100% organic wheatmeal; 75/25: 75% organic wheatmeal and 25% organic olive-pomace; 50/50: 50% organic wheatmeal and 50% organic olive-pomace; 25/75: 25% organic wheatmeal and 75% organic olive-pomace. Different letters within the same matrix indicate statistically significant differences among experimental groups (*p* < 0.05)

Figure 3 shows results on Ni content in feeding substrates, larvae of *T. molitor* and frass. Wheat flour showed a Ni content (~0.5 mg∙kg^−1^ dw) significantly lower (about four-fold) with respect to the other feeding substrates (*p* < 0.0001). In general Ni content is not significantly different between substrates containing different percentages of organic wheatmeal and organic olive-pomace, because these two ingredients showed a similar Ni concentration. No limits for this metal were reported in the 2002/32 EU regulation.

Ni content in larvae increased from ~0.08 mg∙kg^−1^ dw (larvae reared on organic wheat flour) to ~2 mg∙kg^−1^ dw (larvae reared on 25/75 organic wheatmeal/organic olive-pomace). Statistically significant differences in Ni content were observed between larvae reared on different substrates (*p* < 0.0001), except between larvae reared on feeding substrates composed by 100% organic wheatmeal and by 75% organic whetmeal and 25% organic olive-pomace. No statistically significant correlation was found between Ni content in substrates and in larvae (r = 0.6126, *p* = 0.2720); therefore, it seems the Ni content in larvae is not influenced by Ni content in the feeding substrate. Ni showed BAF values next to 1 (from 0.17 to 1.2) (Table 2); therefore, it did not bioaccumulate in the larvae of *T. molitor*. From these results, we can conclude that Ni present in feeding substrate penetrates in the body of larvae, and then it is excreted without bioaccumulation.

Ni showed the lowest concentration in frass derived from 100% wheat flour, whereas the highest content was found in frass derived from 100% organic wheatmeal. Statistically significant differences were evidenced in Ni content between groups (*p* < 0.0001). No statistically significant correlation was found between Ni content in frass and in the feeding substrate (r = 0.5880, *p* = 0.2971).

### 3.4. Arsenic

**Figure 4:**
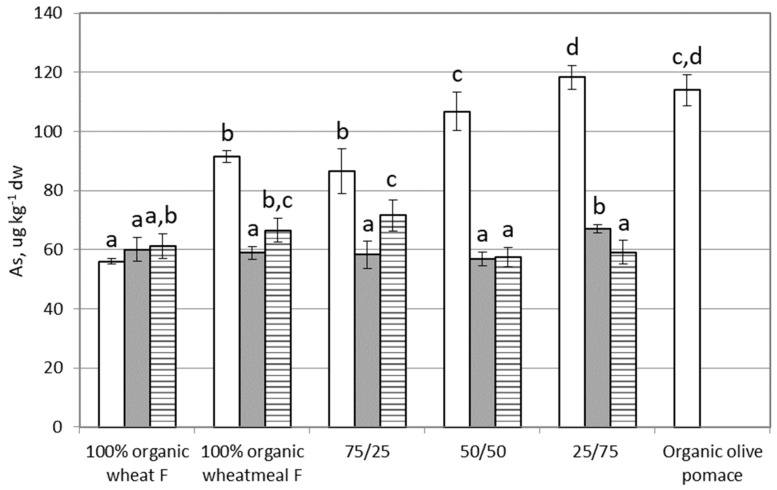
Arsenic content (as µg∙kg^−1^ dw) in feeding substrates and in organic olive-pomace (white bars), *T. molitor* larvae (grey bars), and frass (black and white bars). Each bar represents the mean ± standard deviation of at least three measurements. Feeding substrates: 100% organic wheat flour; 100% organic wheatmeal; 75/25: 75% organic wheatmeal and 25% organic olive-pomace; 50/50: 50% organic wheatmeal and 50% organic olive-pomace; 25/75: 25% organic wheatmeal and 75% organic olive-pomace. Different letters within the same matrix indicate statistically significant differences among experimental groups (*p* < 0.05).

Figure 4 shows results of As content in feeding substrates, larvae of *T. molitor*, and frass. Organic wheat flour feeding substrate showed the lowest As content (~56 µg∙kg^−1^ dw), whereas organic olive-pomace and the feeding substrate composed by 25% organic wheathmeal and 75% organic oliv- pomace showed the highest concentration (~115 µg∙kg^−1^ dw). Statistically significant differences were evidenced between feeding substrates (*p* < 0.0001). Referring the EC limit (2002/32/EC) on undesirable substances in animal feed, the legal limit for As in complete feed is 2 mg∙kg^−1^ (maximum content relative to a feedingstuff with a moisture content of 12%). As content in tested feeding substrates (calculated for a moisture content of 12%) varied from 50 to 100 µg∙kg^−1^, i.e., from 20 to 40-fold lower than the EC limit. Then, these feeding substrates can be considered safe from the point of view of As content.

As content in larvae (from 60 to 67 µg∙kg^−1^) is similar between groups, and only As content in larvae reared on 25% organic wheatmeal and 75% organic olive-pomace is statistically higher then As content in other groups of larvae (*p* = 0.0002). Moreover, no statistically significant correlation was found between As content in feeding substrates and in larvae (r = 0.3884, *p* = 0.5182); therefore, the content of As in larvae is not influenced by As present in the feeding substrate. As showed BAF values ≤ 1 (Table 2), then it did not bioaccumulate (BAF < 1) in the larvae of *T. molitor*. From these results, we can conclude that As present in the feeding substrate penetrates in the body of larvae, and then it was excreted without bioaccumulation.

As in frass varied from 60 to 72 µg∙kg^−1^, and statistically significant higher values were evidenced in frass deriving from feeding substrates composed by 100% organic whetameal and by a mix of organic wheatmeal and organic olive-pomace (75% to 25%), with respect to other frass (*p* = 0.0084). No statistically significant correlation was found between Ni content in frass and in the feeding substrate (r = −0.3053, *p* = 0.6174).

### 3.5. Mercury

**Figure 5:**
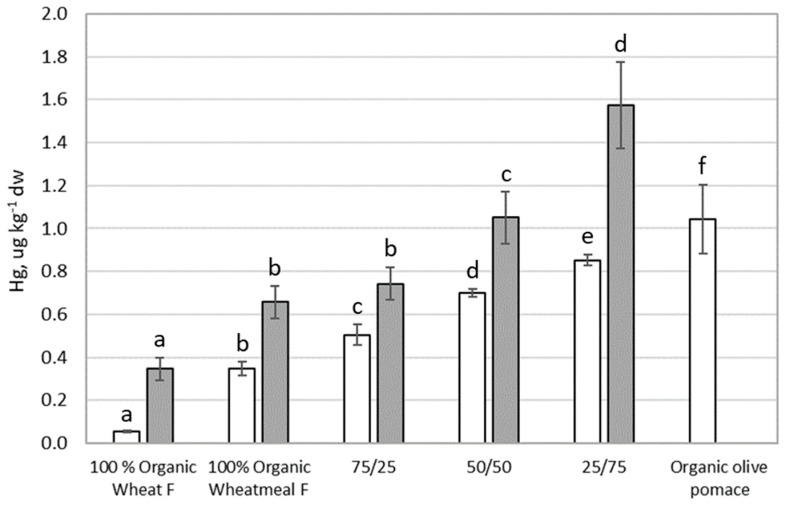
Mercury content (as µg∙kg^−1^ dw) in feeding substrates and in organic olive-pomace (white bars), *T. molitor* larvae (grey bars). Each bar represents the mean ± standard deviation of at least three measurements. Feeding substrates: 100% organic wheat flour; 100% organic wheatmeal; 75/25: 75% organic wheatmeal and 25% organic olive-pomace; 50/50: 50% organic wheatmeal and 50% organic olive-pomace; 25/75: 25% organic wheatmeal and 75% organic olive-pomace. Different letters within the same matrix indicate statistically significant differences among experimental groups (*p* < 0.05).

Figure 5 shows results of Hg content in feeding substrates and larvae of *T. molitor*. Hg showed a very low content in feeding substrates (≤1 µg∙kg^−1^ dw), and statistically significant differences in Hg content were observed between all feeding substrates (*p* < 0.0001); organic wheat flour showed the lowest Hg content, whereas organic olive-pomace showed the highest concentration.

Referring the EC limit (2002/32/EC) on undesirable substances in animal feed, the legal limit for Hg in complete feed is 0.1 mg∙kg^−1^ (maximum content relative to a feedingstuff with a moisture content of 12%). Hg content in tested feeding substrates (calculated for a moisture content of 12%) is <1 µg∙kg^−1^, about three order of magnitude lower than the EC limit. Then, these feeding substrates are absolutely safe from the point of view of Hg content.

Hg content in larvae varied from 0.3 to 1.6 µg∙kg^−1^ dw. Statistically significant differences in Hg content were observed between larvae reared on different substrates *(p* < 0.0001), except between larvae reared on 100% organic wheatmeal and on feeding substrate composed by 75% organic wheathmeal and 25% organic olive-pomace. A statistically significant linear correlation was found between Hg content in feeding substrates and in larvae (r = 0.9493, *p* = 0.0136), highlighting that the content of Hg in larvae is clearly influenced by Hg content in the feeding substrate. The BAF for Hg (Table 2) was from 1.5 to 6.9, indicating a bioaccumulation capacity. These data confirm the bioaccumulation capacity of Hg in organisms, as reported by literature [47,48].

### 3.6. Selenium Health Benefit Values: Mercury Risk Assessments

**Figure 6:**
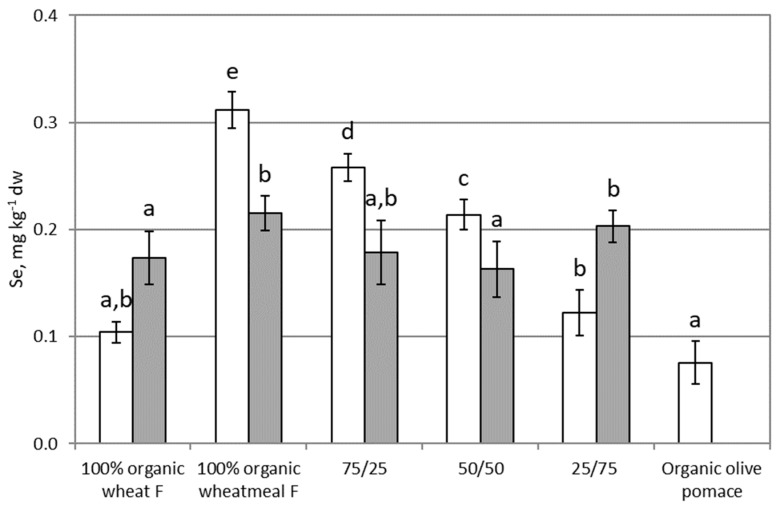
Se content (as mg∙kg^−1^ dw) in feeding substrates and in organic olive-pomace (white bars), *T. molitor* larvae (grey bars). Each bar represents the mean ± standard deviation of at least three measurements. Feeding substrates: 100% organic wheat flour; 100% organic wheatmeal; 75/25: 75% organic wheatmeal and 25% organic olive-pomace; 50/50: 50% organic wheatmeal and 50% organic olive-pomace; 25/75: 25% organic wheatmeal and 75% organic olive-pomace. Different letters within the same matrix indicate statistically significant differences among experimental groups (*p* < 0.05).

Figure 6 shows results on Se content in feeding substrates and larvae of *T. molitor*. Concerning substrates, organic wheat flour and organic olive-pomace showed the lowest Se content (about 0.1 mg∙kg^−1^ dw), whereas organic wheatmeal showed the highest concentration (~0.31 mg∙kg^−1^ dw). By consequence, feeding substrates obtained mixing organic wheatmeal and organic olive-pomace showed a statistically significant decrease in Se content with increasing the percentage of organic olive-pomace (*p* < 0.0001).

The Se content in larvae varied from 0.17 to 0.22 mg∙kg^−1^ dw, and was consistent with literature data [6]. Larvae reared on 100% organic whetameal and on feeding substrate composed by 25% organic wheatmeal and 75% organic olive-pomace showed significantly higher Se content than larvae reared on other feeding substrates (*p* = 0.050). No statistically significant correlation was found between Se content in larvae and in the corresponding feeding substrate (r = 0.3046, *p* = 0.6182). Se is an essential micronutrient, associated to numerous seleno-proteins [49], then its uptake is subjected to a homeostatic regulation, and regulated by the needs of the insect. The BAF value next to 1 (Table 2), showed that Se was not accumulated in the organism, and confirmed its role as an essential element. With respect to Se, no regulation exists for food, since it is an essential element. However, the recommended dietary allowance (RDA) for Se [50] (based on the amount needed to maximize the synthesis of the selenoprotein glutathione-peroxidase) is 55 μg/day, whereas the tolerable upper intake level (UL) for human adults is set at 400 μg/day (based on selenosis as the adverse effect). The Se content found in mealworm larvae, reared on substrates made up of organic wheat milling and olive processing by-products (from 0.057–0.085 mg∙kg^−1^ ww), is comparable to Se content of legumes (such as lentils, chickpeas, beans), dried fruit (such as peanut, pistachios, nuts), or cereals (rice and barley). Therefore, larvae of *T. molitor* are poor in Se content: a portion of 0.6–1 kg ww/day (or 0.25–0.32 kg dw/day) is necessary to reach the RDA. The protective action of Se against Hg is related mainly to the formation of Hg-Se complexes [29], but this mechanism in food contaminated by Hg could reduce the Se quota amount necessary for normal body functions. Therefore, the selenium health benefit value (HBV_Se_) provides a clear instrument to better understand the quote of relative Se available that remains after its Hg-interaction, and to classify the risk posed by consuming these foods. The HBV_Se_ value for healthy food should be >0 [42]—a positive sign would negate risks associated with Hg exposure, whereas negative values are associated with food potentially dangerous for Hg toxicity; the value found in *T. molitor* larvae was 0.17, 0.21, 0.17, 0.16, 0.20 for larvae reared respectively on 100% organic wheat flour, 100% organic wheatmeal, 75% organic whetahmeal + 25% organic olive-pomace, 50% organic whetahmeal + 50% organic olive-pomace, and 25% organic whetahmeal + 75% organic olive-pomace. The HBV_Se_ values next to 0 confirmed the low levels of Se recorded, but indicate that mealworm larvae, reared on substrates made up of organic wheat milling and olive processing by-products, have enough Se to proctect against Hg toxicity, and can be considered a safe food from the point of view of Hg intake.

### 3.7. Heavy Metals Content in T. molitor Larvae and Comparison with Legal Limit for Food

To compare heavy metals content of *T. molitor* larvae with the legal limit for food (EU commission regulation No 1881/2006 of 19 December, 2006 setting maximum levels for certain contaminants in foodstuffs, and amending Regulations No 420/2011 of 29 April, 2011, and No 1006/2015, of 25 June, 2015 as regards maximum levels of inorganic arsenic in foodstuffs), we reported in Table 3 data referred to wet weight.

Cd, Pb, As and Hg levels found in larvae of *T. molitor* were below the respective legal limit (no law limit for Ni). Published data on the presence and content of metals in mealworm larvae or comparable edible insects are rather scarce (and in general in these studies feeding substrates spiked with metals were used), which makes it difficult to compare our results with other studies. Nevertheless, some comparison can be made. The levels of Cd, As and Ni are consistent with those found by Poma et al. [15] in mealworm larvae (mean levels 0.06, <0.03, and <0.28 mg∙kg^−1^, respectively), and lower than those found by Hyun et al. [51] in edible grasshoppers from Korea (Cd 0.02, As 0.12 mg∙kg^−1^) considered as safe for human consumption. Pb content was higher than Pb levels found in Poma et al. [15] (<0.03 mg∙kg^−1^), but lower than Pb content found in edible grasshoppers from Korea (0.73 mg∙kg^−1^) [51].

## 4. Conclusions

The chemical analyses on *T. molitor* larvae, fed, and frass were performed, to assess the chemical risks linked with insect consumption as food and insect-based food products. For this purpose, toxic metals Cd, Pb, Ni, As and Hg were studied. The Se concentration was also determined, to evaluate the risk/benefit value for the human consumption of larvae of *T. molitor* relatively to Hg intake. Concerning feeding substrates, wheat flour resulted in the least contaminated results all considered metals; organic wheatmeal showed the highest concentration of Cd and Pb, whereas organic olive-pomace resulted the largest source of Hg and As. These last two feeding substrates also showed the highest concentration of Ni. In any case, Cd, Pb, As and Hg concentrations found in feeding substrates were below the legal limit of undesirable substances in animal feed (2002/32/EC, no limit are reported for Ni), then we can conclude that tested feeding substrates were safe from the point of view of heavy metal content and can be used to rear insects.

About mealworm larvae, Hg showed the lowest content with respect to other considered metals (from 0.3 to 1.6 µg∙kg^−1^ dw). A statistically significant correlation between metal content in feeding substrates and larvae of *T. molitor* was found only for Hg, which tend to bioaccumulate in insects. Moreover, the BAF next to 1 for Cd, Ni, and As indicated that these metals penetrate in the body of larvae, and then they were exctreted without bioaccumulation. The high levels of Pb in larvae, relatively to Pb content in feeding substrate, suggested that organic carrots (generally rich in Pb content), supplied as water source for larvae, could be responsible of the high Pb content in larvae. When compared to legal limits (EU 420/2011 and EU 1006/2015), the heavy metal content of larvae of *T. molitor* was below the legal limit for Hg, Cd, Pb, and As (no law limit for Ni). Morover, the HBV_Se_ value indicated that the low Se content is sufficient to protect against Hg toxicity, and larvae of *T. molitor* can be considered a safe food from the point of view of Hg intake. Overall, our results indicate that the risk of exposure to metals from the consumption of mealworm larvae is relatively low and in compliance with European Union regulations. Due the scarcity of literature data about toxic metals content in *T. molitor*, this study can improve knowledge in this field, increasing the acceptance and the change in the approach of developed countries with regards to entomophagy.

## Figures and Tables

**Table 1:** Results and certified values of the certified reference material DORM-2 (dog fish muscle), NRC Canada. Data are expressed in mg∙kg^−1^.

Element	Analytical Method	Analytical Results	Certified Values	Δ (%)
Cd	GF-AAS	0.044 ± 0.002	0.043 ± 0.008	+2
Pb	GF-AAS	0.069 ± 0.004	0.065 ± 0.007	+6
Ni	GF-AAS	17.2 ± 0.3	19.4 ± 3.1	−11
As	GF-AAS	17.0 ± 0.7	18 ± 1.1	−5.5
Hg	DMA-1	4.43 ± 0.05	4.58 ± 0.16	+3
Se	GF-AAS	1.41 ± 0.02	1.40 ± 0.09	−1

**Table 2:** Bioaccumulation factor (BAF) for larvae of *T. molitor* reared on tested feeding substrates, calculated on a dry weight basis.

Element	100% Organic Wheat Flour	100% Organic Wheatmeal	75/25 *	50/50 *	25/75 *
Cd	1.7 ± 0.2	0.8 ± 0.1	0.9 ± 0.1	1.4 ± 0.1	1.9 ± 0.2
Pb	34 ± 4	5.2 ± 0.4	6.1 ± 0.5	9.8 ± 0.9	12 ± 1
Ni	0.17 ± 0.04	0.36 ± 0.08	0.43 ± 0.05	0.61 ± 0.08	1.18 ± 0.12
As	1.07 ± 0.07	0.64 ± 0.03	0.67 ± 0.08	0.53 ± 0.04	0.57 ± 0.02
Hg	6.2 ± 1.1	1.9 ± 0.3	1.5 ± 0.2	1.5 ± 0.2	1.8 ± 0.2
Se	1.7 ± 0.3	0.7 ± 0.1	0.7 ± 0.1	0.8 ± 0.1	1.7 ± 0.3

* 75/25: 75% organic wheatmeal and 25% organic olive-pomace; * 50/50: 50% organic wheatmeal and 50% organic olive-pomace; * 25/75: 25% organic wheatmeal and 75% organic olive-pomace.

**Table 3:** Of metals in insects and legal limits (guidelines EU No 1881/2006 and amending regulations No 420/2011 and No 1006/2015), referred to wet weight (mg∙kg^−1^ ww).

Metals	100% Organic Wheat Flour	100% Organic Wheatmeal	75/25	50/50	25/75	Legal Limit (a,b)
Cd	0.011 ± 0.001	0.016 ± 0.002	0.011 ± 0.001	0.008 ± 0.001	0.011 ± 0.002	0.05 (meat) ^a^
Pb	0.066 ± 0.008	0.079 ± 0.005	0.066 ± 0.004	0.063 ± 0.005	0.073 ± 0.003	0.1 (meat) ^a^
Ni	0.030 ± 0.007	0.26 ± 0.05	0.30 ± 0.03	0.47 ± 0.04	0.63 ± 0.04	Not reported
As	0.021 ± 0.001	0.023 ± 0.001	0.022 ± 0.002	0.021 ± 0.001	0.021 ± 0.001	0.20 (rice) ^b^
Hg	0.12 × 10^−3^ ± 0.01 × 10^−3^	0.26 × 10^−3^ ± 0.03 × 10^−3^	0.28 × 10^−3^ ± 0.01 × 10^−3^	0.38 × 10^−3^ ± 0.03 × 10^−3^	0.49 × 10^−3^ ± 0.04 × 10^−3^	0.5 (fish) ^a^

Feeding substrates: 100% organic wheat flour; 100% organic wheatmeal; 75/25: 75% organic wheatmeal and 25% organic olive-pomace; 50/50: 50% organic wheatmeal and 50% organic olive-pomace; 25/75: 25% organic wheatmeal and 75% organic olive-pomace. ^a^ 1881/2006/EU and 420/2011/EU. ^b^ 1006/2015/EU.

## References

[1] Van Huis A., Van Itterbeeck J., Klunder H., Mertens E., Halloran A., Muir G., Vantomme P. (2013). Edible Insects: Future Prospects for Food and Feed Security.

[2] Henry M., Gasco L., Piccolo G., Fountoulaki E. (2015). Review on the use of insects in the diet of farmed fish: Past and future. Anim. Feed Sci. Technol..

[3] Vargas A., Randazzo B., Riolo P., Truzzi C., Gioacchini G., Giorgini E., Loreto N., Ruschioni S., Zarantoniello M., Antonucci M. (2018). Rearing zebrafish on black soldier fly (*Hermetia illucens*): Biometric, histological, spectroscopic, biochemical, and molecular implications. Zebrafish.

[4] Zarantoniello M., Bruni L., Randazzo B., Vargas A., Gioacchini G., Truzzi C., Annibaldi A., Riolo P., Parisi G., Cardinaletti G. (2018). Partial dietary inclusion of *Hermetia illucens* (Black Soldier Fly) full-fat prepupae in zebrafish feed: Biometric, histological, biochemical, and molecular implications. Zebrafish.

[5] Belluco S., Losasso C., Maggioletti M., Alonzi C.C., Paoletti M.G., Ricci A. (2013). Edible insects in a food safety and nutritional perspective: A critical review. Compr. Rev. Food Sci. Food Saf..

[6] Nowak V., Persijn D., Rittenschober D., Charrondiere U.R. (2016). Review of food composition data for edible insects. Food Chem..

[7] Payne C.L.R., Scarborough P., Rayner M., Nonaka K. (2016). A systematic review of nutrient composition data available for twelve commercially available edible insects, and comparison with reference values. Trends Food Sci. Technol..

[8] EFSA Scientific Committee (2015). Scientific opinion on risk profile related to production and consumption of insects as food and feed. EFSA J..

[9] Truzzi C., Giorgini E., Annibaldi A., Antonucci M., Illuminati S., Scarponi G., Riolo P., Isidoro N., Conti C., Zarantoniello M. (2019). Fatty acids profile of black soldier fly (*Hermetia illucens*): Influence of feeding substrate based on coffee-waste silverskin enriched with microalgae. Anim. Feed Sci. Technol..

[10] Bednarska A.J., ZuzannaŚwiątek Z. (2016). Subcellular partitioning of cadmium and zinc in mealworm beetle (*Tenebrio molitor*) larvae exposed to metal-contaminated flour. Ecotoxicol. Environ. Saf..

[11] Lindqvist L. (1992). Accumulation of Cadmium, Copper, and Zinc in Five Species of Phytophagous Insects. Environ. Entomol..

[12] Zhuang P., Zou H., Shu W. (2009). Biotransfer of heavy metals along a soil-plantinsect-chicken food chain: Field study. J. Environ. Sci..

[13] Devkota B., Schmidt G.H. (2000). Accumulation of heavy metals in food plants and grasshoppers from the Taigetos Mountains, Greece. Agric. Ecosyst. Environ..

[14] Gaylor M.O., Harvey E., Hale R.C. (2012). House crickets can accumulate polybrominated diphenyl ethers (PBDEs) directly from polyurethane foam common in consumer products. Chemosphere.

[15] Poma G., Cuykx M., Amato E., Calaprice C., Focant J.F., Covaci A. (2017). Evaluation of hazardous chemicals in edible insects and insect-based food intended for human consumption. Food Chem. Toxicol..

[16] Schiefenhövel W., Blum P., MacClancy J., Henry J., Macbeth H. (2009). Insects: Forgotten and rediscovered as food. Entomophagy among the Eipo, highlands of West New Guinea, and in other traditional societies. Consuming the Inedible.

[17] Johnson D.V. (2010). The contribution of edible forest insects to human nutrition and forest management. Forest Insects as Food: Humans Bite Back.

[18] Mlcek J., Rop O., Borkovcova M., Bednarova M. (2014). A comprehensive look at the possibilities of edible insects as food in Europe: A review. Pol. J. Food Nutr. Sci..

[19] Li L., Zhao Z., Liu H. (2013). Feasibility of feeding yellow mealworm (*Tenebrio molitor* L.) in bioregenerative life support systems as a source of animal protein for humans. Acta Astronaut..

[20] Rumpold B.A., Schlüter O.K. (2013). Potential and challenges of insects as an innovative source for food and feed production. Innov. Food Sci. Emerg. Technol..

[21] Siemianowska E., Kosewska A., Aljewicz M., Skibniewska K.A., Polak-Juszczak L., Jarocki A., Jędras M. (2013). Larvae of mealworm (*Tenebrio molitor* L.) as European novel food. Agric. Sci..

[22] Vijver M., Jager T., Posthuma L., Peijnenburg W. (2003). Metal uptake from soils and soil–sediment mixtures by larvae of *Tenebrio molitor* (L.) (Coleoptera). Ecotoxicol. Environ. Saf..

[23] Oonincx D.G.A.B., Van Broekhoven S., Van Huis A., Van Loon J.J.A. (2015). Feed conversion, survival and development, and composition of four insect species on diets composed of food by-products. PLoS ONE.

[24] Berbel J., Posadillo A. (2018). Review and analysis of alternatives for the valorisation of agro-industrial olive oil by-products. Sustainability.

[25] Borja R., Raposo F., Rincón B. (2006). Treatment technologies of liquid and solid wastes from two-phase olive oil mills. Grasas Y Aceites.

[26] Dermeche S., Nadour M., Larroche C., Moulti-Mati F., Michaud P. (2013). Olive mill wastes: Biochemical characterizations and valorization strategies. Process Biochem..

[27] Fernández-Bolaños J., Rodríguez G., Rodríguez R., Guillén R., Jiménez A. (2006). Extraction of interesting organic compounds from olive oil waste. Grasas Y Aceites.

[28] Ruiz E., Romero-García J.M., Romero I., Manzanares P., Negro M.J., Castro E. (2017). Olive-derived biomass as a source of energy and chemicals. Biofuels Bioprod. Biorefin..

[29] Khan M.A., Wang F. (2009). Mercury–selenium compounds and their toxicological significance: Toward a molecular understanding of the mercury–selenium antagonism. Environ. Toxicol. Chem..

[30] Park K., Mozaffarian D. (2010). Omega-3 fatty acids, mercury, and selenium in fish and the risk of cardiovascular diseases. Curr. Atheroscler. Rep..

[31] Sakamoto M., Yasutake A., Kakita A., Ryufuku M., Chan H.M., Yamamoto M., Oumi S., Kobayashi S., Watanabe C. (2013). Selenomethionine protects against neuronal de-generation by methylmercury in the developing rat cerebrum. Environ. Sci. Technol..

[32] Li X., Yin D., Yin J., Chen Q., Wang R. (2014). Dietary selenium protect against redox-mediated immune suppression induced by methylmercury exposure. Food Chem. Toxicol..

[33] Hu X.F., Eccles K.M., Chan H.M. (2017). High selenium exposure lowers the odds ratios for hypertension, stroke, and myocardial infarction associated with mercury exposure among Inuit in Canada. Environ. Int..

[34] Broekhovenv V.S., Oonincx D.G., Huis V.A., Loon V.J.J.A. (2015). Growth performance and feed conversion efficiency of three edible mealworm species (Coleoptera: Tenebrionidae) on diets composed of organic by-products. J. Insect Physiol..

[35] Cortes Ortiz J.A., Ruiz A.T., Morales-Ramos J.A., Thomas M., Rojas M.G., Tomberlin J.K., Yi L., Han R., Giroud L., Jullien R.L., Dossey A.T., Morales-Ramos J.A., Rojas M.G. (2016). Insect mass production technologies. Insects as Sustainable Food Ingredients.

[36] Dreassi E., Cito A., Zanfini A., Materozzi L., Botta M., Francardi V. (2017). Dietary fatty acids influence the growth and fatty acid composition of the yellow mealworm *Tenebrio molitor* (Coleoptera: Tenebrionidae). Lipids.

[37] Osimani A., Milanović V., Cardinali F., Garofalo C., Clementi F., Ruschioni S., Riolo P., Isidoro N., Loreto N., Galarini R. (2018). Distribution of transferable antibiotic resistance genes in laboratory-reared edible mealworms (*Tenebrio molitor* L.). Front. Microbiol..

[38] Illuminati S., Annibaldi A., Truzzi C., Scarponi G. (2014). Recent Temporal Variations of Trace Metal Content in an Italian White Wine. Food Chem..

[39] Morgano M.A., Milani R.F., Perrone A.A.M. (2015). Determination of total mercury in sushi samples employing direct mercury analyser. Food Anal. Methods.

[40] Annibaldi A., Truzzi C., Carnevali O., Pignalosa P., Api M., Scarponi G., Illuminati S. (2019). Determination of Hg in farmed and wild atlantic bluefin tuna (*Thunnus thynnus* L.) muscle. Molecules.

[41] Van der Fels-Klerx H.J., Camenzuli L., Van der Lee M.K., Oonincx D.G.A.B. (2016). Uptake of cadmium, lead and arsenic by *Tenebrio molitor* and *Hermetia illucens* from contaminated substrates. PLoS ONE.

[42] Ralston N.V.C., Ralston C.R., Raymond L.J. (2016). Selenium health benefit values: Updated criteria for mercury risk assessments. Biol. Trace Elem. Res..

[43] Daniel W.W., Cross C.L. (2013). Biostatistics: A Foundation for Analysis in the Health Sciences.

[44] (2018). Statgraphics Centurion 18 Software. http://www.statgraphics.com/centurion-xviii.

[45] Zwolak A., Sarzyńska M., Szpyrka E., Stawarczyk K. (2019). Sources of Soil Pollution by Heavy Metals and Their Accumulation in Vegetables: A Review. Water Air Soil Pollut..

[46] Codling E.E., Chaney R.L., Green C.E. (2015). Accumulation of lead and arsenic by carrots grown on lead-arsenate contaminated orchard soils. J. Plant Nutr..

[47] Boudou A., Ribeyre F. (1997). Metal Ions in Biological Systems, Vol. 34: “Mercury and its Effects on Environment and Biology”. Met. Based Drugs.

[48] Kidd K., Clayden M., Jardine T., Liu G., Cai Y., O’Driscoll N. (2012). Bioaccumulation and biomagnification of mercury through food webs. Environmental Chemistry and Toxicology of Mercury.

[49] Reeves M.A., Hoffmann P.R. (2009). The human selenoproteome: Recent insights into functions and regulation. Cell. Mol. Life Sci..

[50] U.S. Department of Health & Human Services the Office of Dietary Supplements (ODS) of the National Institutes of Health (NIH) Selenium Fact Sheet for Health Professionals. https://ods.od.nih.gov/factsheets/Selenium-HealthProfessional/.

[51] Hyun S.H., Kwon K.H., Park K.H., Jeong H.C., Kwon O., Tindwa H., Han Y.S. (2012). Evaluation of nutritional status of an edible grasshopper, Oxya Chinensis Formosana. Entomol. Res..

